# Is ^99m^Tc bone scintigraphy necessary in the preoperative workup for patients with cT1N0 subsolid lung cancer? A prospective multicenter cohort study

**DOI:** 10.1111/1759-7714.13752

**Published:** 2020-11-19

**Authors:** Hang Li, Ting Ye, Nan Li, Guozhan Xia, Bin Li, Yang Zhang, Hong Hu, Yihua Sun, Yawei Zhang, Jiaqing Xiang, Dongchun Ma, Yuan Weng, Shilei Liu, Chunyi Jia, Bin Qian, Yajia Gu, Yuan Li, Shaoli Song, Haiquan Chen

**Affiliations:** ^1^ Departments of Thoracic Surgery and State Key Laboratory of Genetic Engineering Fudan University Shanghai Cancer Center Shanghai China; ^2^ Institute of Thoracic Oncology Fudan University Shanghai China; ^3^ Department of Oncology Shanghai Medical College, Fudan University Shanghai China; ^4^ Department of Nuclear Medicine Fudan University Shanghai Cancer Center Shanghai China; ^5^ Department of Thoracic Surgery Anhui Chest Hospital Hefei China; ^6^ Department of Thoracic Surgery Affiliated Hospital of Jiangnan University Wuxi China; ^7^ Department of Thoracic Surgery Henan Cancer Hospital Zhengzhou China; ^8^ Department of Thoracic Surgery Jilin Cancer Hospital Changchun China; ^9^ Department of Thoracic Surgery Jiang Du People's Hospital Jiangdu China; ^10^ Department of Radiology Fudan University Shanghai Cancer Center Shanghai China; ^11^ Department of Pathology Fudan University Shanghai Cancer Center Shanghai China

**Keywords:** Bone scintigraphy, cT1N0 subsolid lung cancer, preoperative workup

## Abstract

**Background:**

^99m^Tc bone scintigraphy (BS) is still the most common approach for the evaluation of bone metastasis in China. The purpose of this study was to investigate the necessity of BS as part of a routine preoperative workup for patients with cT1N0 subsolid lung cancer.

**Methods:**

This was a prospective multicenter clinical trial (NCT03689439). Patients with cT1N0 subsolid nodules who were candidates for surgical resection were consecutively enrolled into the study. BS was performed preoperatively. The surgical plan could be changed if a positive result was detected. The primary endpoint was the incidence rate of the surgical plan being changed because of positive BS results. The secondary endpoint was the rate of positive BS findings and the rate of related complications.

**Results:**

From November 2018 to July 2019, 691 patients were enrolled into the study. None of the patients had positive BS results and no surgical plans were changed by BS findings. There were 222 male and 469 female patients. The average age was 54.8 ± 3.7 years old. The average tumor diameter was 14.9 ± 4.2 mm. There were 282 patients with pure GGO nodules and 409 with part‐solid nodules. A total of 470 patients had a single nodule, while 221 patients had multifocal lesions. The number of patients whose pathological diagnosis was invasive adenocarcinoma, minimally invasive adenocarcinoma, adenocarcinoma in situ and mucinous adenocarcinoma was 357, 293, 32 and nine, respectively. The number of patients who underwent lobectomy, segmentectomy and wedge resection was 234, 199 and 258, respectively.

**Conclusions:**

^99m^Tc bone scintigraphy is unnecessary in the preoperative workup for patients with cT1N0 subsolid lung cancer.

**Key points:**

**Significant findings of the study:**

In this prospective study of 691 patients with cT1N0 subsolid lung cancer, no surgical plans were affected by positive bone scan findings.

**What this study adds:**

We suggest physicians consider canceling BS from preoperative workup for cT1 subsolid lung cancer patients.

Clinical trial registry number: NCT03689439.

## Graphical abstract

In this prospective study of 691 patients with cT1N0 subsolid lung cancer, no surgical plans were affected by positive bone scan findings. We suggest physicians consider canceling BS from preoperative workup for cT1 subsolid lung cancer patients.

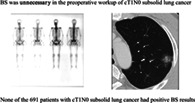

## Introduction

The skeleton is a common metastatic site for advanced lung cancer, and patients with bone metastases (BM) might lose the opportunity for surgery with a significantly worse prognosis.^1^ Although PET‐CT is widely applied for the detection of distant metastases,^2^, ^3^ ^99m^Tc bone scintigraphy (BS) is still recognized as an important method for preoperative detection and postoperative follow‐up of BM in various malignancies.^4^, ^5^, ^6^ Considering the advantages in accessibility, BS is still the most common modality for detecting BM in the preoperative staging procedure in China.^7^

However, our previous study showed that few BM were found in apparently early stage non‐small cell lung cancer (NSCLC) cases in the preoperative staging procedure. In this study, no BM was found in ground‐glass opacity (GGO) type lung cancer and the incidence of BM in cT1 lung cancer was 0.94% (7 out of 739).^8^ Accordingly, the issue of whether to perform BS preoperatively in all NSCLC patients is controversial.^9^, ^10^, ^11^, ^12^ Considering the disadvantages of BS, including additional cost of medical resources and radiation exposure, the aim of this study was to investigate the necessity of BS as a routine preoperative workup for patients with cT1N0 subsolid lung cancer.

## Methods

### Eligibility criteria

The criteria for inclusion into this trial were: (i) Patients aged between 18 and 80 years; (ii) patients with an ECOG score of less than two; (iii) no bone related symptoms; (iv) diameter of nodules was less than, or equal to, 3 cm; (v) no contraindication for surgery; (vi) preoperative serum carcinoembryonic antigen (CEA) level less than 5 ng/uL; and (vii) a final pathological diagnosis of NSCLC. The exclusion criteria were: (i) Previous history of malignancy; and (ii) related bone disease which can cause osteogenesis or osteolysis. The primary endpoint was the incidence rate of the surgical plan being changed because of positive BS results. It was defined as the number of patients whose treatment strategy was changed by positive BS results divided by the total number of patients. The secondary endpoint was the rate of positive BS results and the rate of procedural complications.

### Sample size estimation

Our previous study showed that the incidence of BM in cT1N0 lung cancer patients was 0.94%.^8^ We hypothesized that if the incidence rate of patients with cT1N0 subsolid lung cancer who had their surgical plan changed because of positive BS results was 0.5% (P0), then preoperative BS examination would be unnecessary in these patients; if the incidence rate of patients who had their surgical plan changed because of positive BS results was 2% (P1), then the preoperative BS examination would be regarded as a routine preoperative workup for cT1N0 radiological subsolid lung cancer. Null hypothesis: H0: *P* ≤ P0; alternative hypothesis: HA: *P* ≥ P1. α = 0.05, 1‐β = 0.9. Simon's optimal two‐stage design showed that 206 patients were the minimum sample size in stage one and in stage two, and 346 patients were the minimum sample size. In total, this trial necessitated at least 552 patients to determine the possibility of canceling BS in the preoperative workup. If none of the 552 patients with cT1 radiological subsolid lung cancer had BM, then preoperative BS examination would be unnecessary.

### Statistical analysis

Baseline characteristics of patients are reported as number (%) for categorical variables. All statistical analyses were performed using Microsoft Office Excel 2007 (Microsoft, Redmond, WA), PASS version 11.0 (NCSS, Kaysville, UT) and SPSS version 18.0 (IBM, Chicago, IL).

### Clinical practice

Before surgery, two chest radiologists evaluated the thin‐section computed tomography (CT) images on lung window settings (window width, 1600 HU; window level, −600 HU; width and interval, 1.0 and 1.0 mm) to confirm the radiological characteristics of the nodules. The maximum diameter of the nodules was measured on the single largest axial dimension measured on a lung window and an edge‐enhancing filter was recorded for solid component and whole nodule size. Subsolid nodules were diagnosed depending on the presence of GGO. If the nodules were not 100% solid or contained any component of GGO, they were defined as subsolid nodules. In the subsolid nodules, a pure ground‐glass nodule (pGGN) was defined as a nodule without any solid component, and part solid nodules (PSN) as a lung lesion with both GGO and solid component. GGO was defined as a nonspecific finding on CT scans that indicates a partial filling of air spaces in the lungs by exudate or transudate, as well as interstitial thickening or partial collapse of lung alveoli.

BS would be performed no more than one week before surgery. The BS examination protocol was followed in accordance with the European Association of Nuclear Medicine guidelines.^13^ If BS results showed patients with suspicious bone metastasis, contrast CT or contrast magnetic resonance imaging (MRI) would be performed on the patients to confirm the results. Written informed consent was received from all enrolled patients.

Clinical and pathological characteristics, including age, sex, tumor size, smoking history, pathological subtype, TNM stage, surgical approach and extent were collected using the case report form (CRF). Postoperative pathological diagnosis was made according to the 2015 World Health Organization (WHO) Classification of Tumors of the Lung, Pleura, Thymus and Heart.^14^ Lung adenocarcinoma was classified according to the 2011 IASLC/ATS/ERS classification of lung adenocarcinoma as adenocarcinoma in situ (AIS), minimal invasive adenocarcinoma (MIA), invasive adenocarcinoma (IAD).^15^ Pathological staging was according to the eighth edition of the TNM classification of lung cancer.^16^

This prospective, multicenter cohort study was conducted in six medical centers, including Fudan University Shanghai Cancer Center, Anhui Chest Hospital, Affiliated Hospital of Jiangnan University, Henan Cancer Hospital, Jilin Cancer Hospital and Jiang Du People's Hospital. This study was approved by the Institutional Review Boards of the six medical centers, respectively. This trial was also registered in Clinicaltrials. gov (BSNTG, NCT03689439).

## Results

From November 2018 to July 2019, 691 patients were enrolled into the study, including 222 male and 469 female patients. None of the patients had positive BS results and no surgical plans were changed as a result of the BS findings. Abnormal results were found in 36 patients, including 25 changes caused by bone fracture which were confirmed by the medical history of the patients. A total of 11 patients underwent contrast CT and contrast MRI of relevant parts of bone and were excluded from having bone metastasis by the radiologists.

The average age of patients in the study was 54.8 ± 3.7 years (ranging from 26 to 81 years old). There were 144 patients with a smoking history, while 547 were never smokers. The average size of all lesions was 14.9 ± 4.2 mm (ranging from 5 to 30 mm). With regard to the GGO component, 282 patients had pure GGO lesions, while 409 patients had part‐solid nodules. A total of 470 patients had a single nodule, while 221 patients had multifocal lesions.

A total of 635 patients received VATS surgery and 56 patients received muscle‐sparring incision thoracotomy. The number of patients who underwent wedge resection, segment resection and lobectomy were 258, 199 and 234, respectively. The average hospital stay time was 4.3 ± 1.2 days (ranging from two to 15 days). A total of 32 patients had adenocarcinoma in situ (AIS), 293 had minimally invasive adenocarcinoma (MIA), and 366 patients had invasive adenocarcinoma, including nine patients with mucinous adenocarcinoma. The number of patients with stage 0, IA1, IA2, IA3 and IB were 27, 268, 344, 46 and six, respectively (Table 1).

**Table 1:** Clinical and pathological characteristics of enrolled patients

Characteristics	N	%
Age (years)	54.8 ± 3.7 (26–81)	
Tumor size (mm)	14.9 ± 4.2 (5–30)	
Hospital stay (days)	4.3 ± 1.2 (2–15)	
Sex		
Male	222	32.1
Female	469	67.9
Smoking history		
Smoker	144	20.8
Nonsmoker	547	79.2
GGO component		
Pure GGO	282	40.8
Part‐solid nodules	409	59.2
Single or multifocal		
Single nodule	470	68.0
Multifocal nodules	221	32.0
Surgical approach		
Open	56	8.1
VATS	635	91.9
Surgical extent		
Wedge resection	258	37.3
Segmentectomy	199	28.8
Lobectomy	234	33.9
Pathology		
Adenocarcinoma in situ	32	4.6
Minimally invasive adenocarcinoma (MIA)	293	42.4
Invasive adenocarcinoma	357	51.7
Mucinous adenocarcinoma	9	1.3
Pathological TNM stage		
0	27	3.9
IA1	268	38.8
IA2	344	49.8
IA3	46	6.7
IB	6	0.9

GGO, ground‐glass opacity; VATS, video‐assisted thoracoscopic surgery.

## Discussion

Precise staging of NSCLC patients is essential because it determines the subsequent therapeutic strategy. One of the most frequent hematogenous metastatic sites of NSCLC is the skeleton. Bone metastasis has been reported to decrease the opportunity of surgery for patients and worsen the survival benefit.^17^, ^18^, ^19^ Bone scintigraphy is recognized as the most sensitive method of detecting bone structure changes, including metastasis, in a series of diseases.^20^ BS is the most used screening procedure, and if the result is positive, contrast CT scan or enhanced MRI is performed to confirm the findings.

In western countries, a fluorodeoxyglucose positron emission tomography/ computed tomography (FDG‐PET/CT) scan is used widely as the routine preoperative evaluation procedure for cT1N0 NSCLC. Considering the accessibility, BS is still the most common modality for detecting BM in China.^7^

In the last decade, a growing number of radiological subsolid lung cancers are diagnosed every year in China. The majority of these cases have been proven to be early stage lung cancer without any metastasis, even though the pathological result was invasive adenocarcinoma. In these patients, detection of bone metastasis was also recommended as the guideline. Irrespective of whether BS or FDG‐PET/CT was performed, both methods were more invasive than the other preoperative workup, with safety issues due to the radiation exposure. Thus, we carried out this trial to evaluate the possibility that BS should be removed from the preoperative workup for cT1N0 radiological subsolid lung cancer.

A total of 409 patients, which accounts for 59.2% of this cohort, had part‐solid lesions. Meanwhile, 396 patients, which accounts for 57.3% of the patients enrolled in the study, had a tumor over 1 cm in diameter. Therefore, the enrolled patients were not only patients with extremely early stage lung cancer. Considering that the surgical plan was not adjusted following positive BS results in any of these patients, BS was unnecessary in the preoperative workup for cT1N0 subsolid lung cancer.

The limitation of this study was that nearly 50% patients in our cohort were diagnosed with AIS/MIA after surgery. We were aware that the possibility of bone metastasis for these patients was extremely low. However, when the patients were detected as having subsolid nodules in the lung, we could not tell if they had AIS/MIA or invasive adenocarcinoma. These patients were not excluded from this study because we intended to investigate the possibility of canceling BS in all patients with cT1 subsolid nodules.

To our knowledge, this is the first multicenter prospective clinical trial evaluating the necessity of performing BS in cT1N0 subsolid lung cancer. It revealed that the incidence rate of patients whose surgical plan was adjusted because of positive BS results was fairly low. Taking into account the additional cost of medical resources and radiation exposure of patients, we suggest that physicians should consider canceling BS from the preoperative workup for cT1 subsolid lung cancer patients.

## Disclosure

We declare that no benefits in any form have been received or will be received from a commercial party related directly or indirectly to the subject of this article. We also declare that we have no conflict of interest in connection with this paper.
